# Isolation of *Candida africana* in oral candidiasis: First report among cancer patients in Iran 

**DOI:** 10.18502/CMM.6.2.2695

**Published:** 2020-06

**Authors:** Ensieh Lotfali, Masoud Mardani, Sara Abolghasemi, David Darvishnia, Mohammad Mahdi Rabiei, Reza Ghasemi, Azam Fattahi

**Affiliations:** 1 Department of Medical Parasitology and Mycology, School of Medicine, Shahid Beheshti University of Medical Sciences, Tehran, Iran; 2 Infectious Diseases and Tropical Medicine Research Center, Shahid Beheshti University of Medial Sciences, Tehran, Iran; 3 Student Research Committee, School of Medicine, Shahid Beheshti University of Medical Sciences, Tehran, Iran; 4 Center for Research and Training in Skin Diseases and Leprosy, Tehran University of Medical Sciences, Tehran, Iran

**Keywords:** Cancer, *Candida africana*, Oral candidiasis

## Abstract

**Background and Purpose:**

Oropharyngeal candidiasis (OPC) is a fungal infection of the oral cavity caused by the members of *C. albicans* complex. Although *C. africana*, as a part of the complex, is considered to be mostly responsible for the development of vulvovaginal candidiasis, it may be associated with a wider clinical spectrum.

**Case report:**

This report described two cases diagnosed with oral candidiasis during the receipt of treatment for malignancies. Conventional and molecular tests were performed on the samples collected from the patients’ oral cavities. The test results revealed *C. africana* as the causative agent of oral candidiasis. Furthermore, in vitro antifungal susceptibility test indicated the full susceptibility of all *C. africana* isolates to caspofungin. However, the data were also suggestive of the resistance against fluconazole and amphotericin B. Caspofungin was used as the main antifungal agent for the treatment of oral candidiasis, resulting in the improvement of thrush in patients. The resistance of *C. africana* to fluconazole and amphotericin B suggests the necessity of performing in vitro susceptibility testing on the isolates for the selection of appropriate antifungal agents.

**Conclusion:**

As the findings indicated, the achievement of knowledge regarding *C. africana* as an emerging non-albicans Candida species and its antifungal susceptibility profile is crucial to select antifungal prophylaxis and empirical therapy for oral candidiasis in cancer patients undergoing chemotherapy.

**None:**

non

## Introduction

Oropharyngeal candidiasis (OPC) is a Candida species-induced fungal infection affecting the oral cavity. The OPC can present with several symptoms, such as angular cheilitis, thrush (pseudomembranous), and erythematous lesion [ 1 ]. The prevalence of invasive fungal infections has an increasing rate, especially in patients with hematological malignancies, such as leukemia and lymphoma [ 2 ]. Moreover, the patients subjected to chemotherapy are at a risk of mucosal integrity destruction and entrance of the colonized organism to the bloodstream. Therefore, the colonization of the oropharyngeal mucosa with* Candida* species is considered a risk factor for invasive candidiasis infections [ 3 ].

*Candida albicans* is the most common etiology of OPC. *Candida albicans* complex consists of *C. albicans*, *C. dubliniensis*, and *C. africana* [ 4 ]. Among the members of this complex, *C. africana* can be frequently colonized in the human vaginal mucosa. However, this species can also be responsible for a great number of serious diseases affecting other human organs [ 5 ]. Herein, we presented two cases of OPC due to *C. africana* in two 46- and 77-year-old men with acute myeloid leukemia and metastatic gastric adenocarcinoma, respectively, who were admitted to Ayatollah Taleghani Hospital in Tehran, Iran. To the best of our knowledge, this is the first report of *C. africana* in patients with cancer.

## Case report

**Case 1**

A 46-year-old man diagnosed with acute myeloid leukemia received 7+3 regimen, including cytarabine (200 mg/m^2^) and idarubicin (12 mg/m^2^) for induction chemotherapy, in addition to fluconazole (400 mg daily), ciprofloxacin (500 mg twice a day), and acyclovir (400 mg twice a day) for antimicrobial prophylaxis during infliction with neutropenia [ 6 ]. He had no history of diabetes mellitus. After 8 days, he presented with fever and neutropenia (absolute neutrophil count: 120/µl).

Physical examination was unremarkable, and vital signs other than fever were stable. Meropenem (2 g; three times a day) was started as an empirical anti-pseudomonas agent for the treatment of fever with an unknown source in neutropenic patients. The development of fever in a patient with an absolute neutrophil count of < 500/µ can be defined as neutropenic fever [ 6 ]. Laboratory evaluation revealed anemia (hemoglobin=8 g/dl) and thrombocytopenia with a platelet count of 7,000 per microliter, blood urea nitrogen (BUN) of 24 mg/dl, and creatinine (Cr) level of 0.8 mg/dl. However, other laboratory tests were normal. Furthermore, the results of the blood and urine cultures were negative. After 4 days, fever still persisted; therefore, liposomal amphotericin (3 mg/kg) was empirically prescribed following the guidelines related to the treatment of infection in cancer patients [ 6 ]. Nevertheless, all the secondary evaluations for persistent fever, such as galactomannan and pulmonary and sinus computed tomography scans, were normal in the neutropenic patient after 4 days.

**Figure 1:**
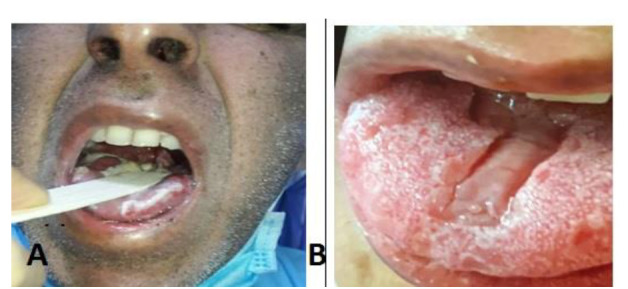
Appearance of white confluent plaques on the tongue in case 1 (A) and case 2 (B)

The patient had a new complaint of dysphagia. The symptoms of severe mucositis (Health World Organization (WHO] grade 3) with oral thrush (Figure 1 A), and swollen uvula were detected in physical examination. After 3 days of receiving liposomal amphotericin B, the signs and symptoms had no improvement. Nasopharyngeal CT scan was unremarkable. As a result, endoscopy was requested; however, it was delayed due to thrombocytopenia. Caspofungin was substituted for liposomal amphotericin and daily administered at a dose of 70 mg at first and then 50 mg. In addition, the sampling of oral thrush was performed for more evaluations. After 3 days, fever and thrush improved. The treatment was continued for 14 days in order to complete the duration of the treatment of OPC and possible esophagitis.

**Case 2**

A 77-year-old man, a known case of metastatic gastric adenocarcinoma diagnosed 7 months earlier, presented with fever and dyspnea. Physical examination was unremarkable unless reduced breath sounds in the lower level of both lungs and evidence of ascites. He had stable vital signs. Pleural effusion was detected in the patient CT scan. He had received five courses of chemotherapy, including cisplatin (75 mg/m^2^) and fluorouracil (750g/m^2^), the latter of which was performed 2 weeks before his referral. The first course of chemotherapy was complicated by oral thrush and mucositis; therefore, he received fluconazole (400 mg daily) for secondary prophylaxis after each course. The patient had no history of diabetes mellitus. Blood and urine cultures were sent for diagnostic workup for fever. Abdominal sonography revealed ascites without any abscess.

The ascites analysis revealed the white blood cell (WBC) count of 800 /µl and polymorphonuclear leukocyte rate of 80%. In addition, the results of complete blood count tests showed the WBC of 6000 /µl, hemoglobin level of 10 g/dl, platelet count of 130,000 /µl BUN of 20 mg/dl, and Cr of 1.02 mg/dl. Piperacillin-tazobactam (4.5 g) was started and administered three times daily due to the impression of spontaneous bacterial peritonitis. 

Three days after the onset of treatment, fever improved; however, severe mucositis (WHO grade 3) with oral lesions developed consistent with thrush (Figure 1 B). The patient received nystatin oral suspension (5 mL; 500,000) four times a day; however, it did not respond. Therefore, nystatin was discontinued, and fluconazole (200 mg) was started twice a day. Three days later, the sampling of the lesion was performed due to treatment failure, and fluconazole was changed with caspofungin (70 mg) at the first dose and then 50 mg daily because of severe odynophagia. Thrush improved in the patient 4 days later. Treatment was continued for 14 days over OPC therapy [ 7 ].

**Laboratory examination**

Sampling of two oral thrush cases was performed and sent for mycological examination to the Department of Mycology, School of Medicine, Shahid Beheshti University of Tehran, Iran. Two sterile swabs were used to collect samples from the oral cavity of the two patients. One swab was used for microscopic examination, and the other one was subjected to fungal culture on Sabouraud dextrose agar with chloramphenicol (Merck, Germany) and then incubated at 30°C for 24 h. Oral thrush was confirmed by the observation of pseudohyphae and budding yeast cells in direct microscopic examination. The produced cream-colored colonies on culture, which were mucoid and smooth, were indicative of* Candida* species colony. 

DNA was extracted from the 24-hour fresh colony culture of the two isolates using a method that was previously described [ 8 ]. Then, the ITS regions of the ribosomal DNA gene of the isolates were amplified by universal fungal primers, namely ITS1 (5´- TCCGTAGGTGAACCTGCGG-3´) and ITS4 (5´-TCCTCCGCTTATTGATATGC-3´). The polymerase chain reaction (PCR) product was subjected to sequencing for identification (Bioneer, Korea). The obtained sequences were compared with the similar sequences in the open-access NCBI database (http://blast.ncbi.nlm. nih.gov/Blast.cgi). The alignment of the obtained sequence with those of the BLAST revealed a 98.9% identity with *C. albicans*. 

**Figure 2:**
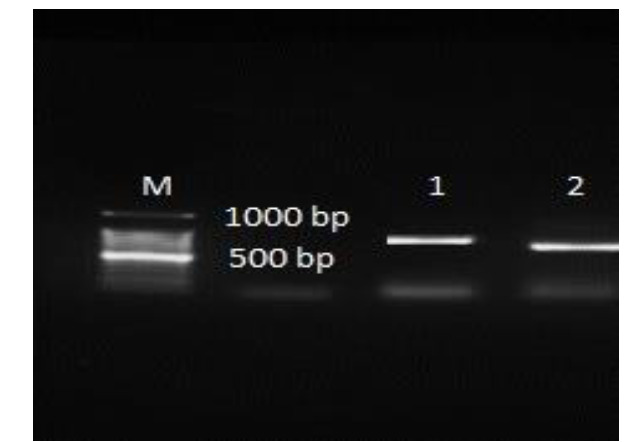
Discrimination of *Candida africana* from *C. albicans*, using the *HWP* primers (*C. africana* (700 bp]); lanes 1, 2, and M) molecular size marker (100 bp)

*Candida albicans* complex consists of *C. albicans*, *C. dubliniensis*, and *C. africana*, which were differentiated using the hyphal wall protein1 (HWP1) gene with primers HWP1-F5′-GCTACCACTTCAGAATCATCATC-3′and HWP1-R5′ GCACCTTCAGTCGTAGAGACG-3′. The complex was differentiated based on the size of the revealed bands, including *C. africana* (700 bp), *C. albicans* (941 bp), and *C. dubliniensis* (569 bp) (Figure 2) [ 9 ]. For the confirmation of species identity, the PCR products of the two isolates of *C. africana* were applied for accurate identification by sequencing (Bioneer, Korea). The alignment of the obtained sequence with those of the BLAST revealed a 98.98% identity with *C. africana* (strain S57AF), which was recorded with the accession number of KY238038.1. It showed high homology (99.85%) to *C. africana* (isolate 241) with the sequence ID of MG434686.1. 

Antifungal susceptibility testing was performed using the broth micro-dilution method based on the Clinical and Laboratory Standards Institute document M27-A3/S4 (Table 1) [ 10 ].

**Table 1:** Results of antifungal susceptibility testing of the two isolates of *Candida africana*

Antifungal	Case 1	Case 2	MIC range (𝜇g/mL)
Fluconazole	MIC: 4 µg/mL	MIC: 16 µg/mL	S: ≤2, I: 4, R: ≥8
Amphotericin	MIC: 4 µg/mL	MIC: 0.25 µg/mL	S: ≤1, R: ≥1
Caspofungin	MIC: 0.0625 µg/mL	MIC: 0.125 µg/mL	S: ≤0.25, I: 0.5. R: ≥1

S: Susceptible, I: Intermediate, R: Resistant, MIC: minimum inhibitory concentration

## Discussion

The present report described two rare cases of oral candidiasis caused by *C. africana* in patients with cancer. There are many studies indicating that *C. africana* can be responsible for vulvovaginal candidiasis [ 5 , 11 ]. Nnadi et al. collected two *C. africana* isolates from patients with vulvovaginal inflammation and reported that all *C. africana* isolates were susceptible to amphotericin B, fluconazole, and caspofungin [ 11 ]. In line with Nnadi et al., Romeo et al. suggested that *C. africana* isolates were susceptible to the commonly used antifungal agents [ 5 ]. In another study, Sharifynia et al. reported that *C. africana* in patients with respiratory infections was resistant to amphotericin B and fully susceptible to fluconazole and caspofungin [ 12 ].

There is only one study reporting *C. africana*-induced oral candidiasis in HIV-infected patients. In the mentioned study, all of the cases (n=4) were susceptible to amphotericin B, caspofungin, and fluconazole [ 13 ]. In our study, the samples were collected from two patients with cancer who developed oral candidiasis due to *C. africana*. According to the epidemiological and clinical data, *C. africana* has a worldwide distribution. This species has been identified in patients residing in Italy, Spain, Poland, the United Kingdom, Chile, Senegal, and Nigeria. This species particularly colonizes and infects human vagina. However, there also some reports regarding the etiologic role of this species in the development of some life-threatening diseases, such as candidemia in adults and renal diseases in newborns. Nonetheless, the clinical significance of *C. africana* remains unclear. The worldwide distribution of *C. africana* and case reports of serious infections warrant the implementation of more studies in the future [ 14 ].

The colonization in the cases presented in this study may have occurred in the hospital from an unknown origin or as a result of contact with vaginal secretion before admission. In the current study, fluconazole was used as a prophylactic agent to protect the patients against invasive fungal infections. In addition, antifungal susceptibility test was performed for caspofungin, fluconazole, and amphotericin B. In agreement with a previous study [ 15 ], two isolates were found to be fully susceptible to caspofungin. 

Although the resistance of* Candida* species to echinocandins has increased, this antifungal agent has remained the preferred first-line class of drug for invasive candidiasis. Moreover, most of* Candida* species are susceptible to this antifungal agent [ 16 ]. However, the are also reports regarding breakthrough infections due to resistant Candida species because of the extensive use of echinocandins to prevent or treat invasive fungal infections [ 17 ].

In contrast to all aforementioned studies [ 5 , 11 - 13 ], our data demonstrated resistance to fluconazole. This resistance to fluconazole can be explained by prior exposure to fluconazole that could increase the colonization of fluconazole-resistant* Candida* species in patients with cancer [ 18 ]. However, in none of the previous studies, prophylactic regimen was indicated [ 5 , 11 - 13 ].

In the current study, one of the isolates was found to be resistant to amphotericin B. This finding could be justified with a previous study demonstrating that the yeasts isolated from the patients undergoing chemotherapy had significantly higher MICs to amphotericin B than the colonizing isolates recovered from immunocompetent patients [ 19 ].

## Conclusion

Oral candidiasis is one of the major complications in patients with malignancies. Mucositis with odynophagia may also occur in patients receiving chemotherapy, which is indistinguishable from esophagitis. The OPC is a symptom that is predictive of the involvement of the esophagus. Endoscopy and sampling could be used for diagnostic purposes; however, they were frequently postponed due to patient conditions, such as bleeding, critical illness, and thrombocytopenia [ 19 ]. In case 1, the patient had esophagitis symptoms, but the diagnosis was not proved because of the low level of platelet in this patient. 

This study was the first report concerning the isolation of *C. africana* from thrush in patients with malignancies in Iran. The acquisition of knowledge regarding the emerging non-albicans* Candida* species (responsible for oral candidiasis) and their antifungal susceptibility is necessary to decide on the antifungal prophylaxis and empirical therapy of oral candidiasis and even invasive infections in the cancer patients undergoing chemotherapy.

## Author’s contribution

E. L., M. M., and S. A. developed the concept and designed the study. D. D. performed sample and data collection. E. L. and A. F. carried out laboratory examinations, as well as data analysis and interpretation. M. M. R. and R. G. H. performed the literature search and prepared the first draft of the manuscript. E. L. and S. A. carried out the final revisions of the manuscript.

## Conflicts of interest

There are no conflicts of interest.

## Financial disclosure

This study was financially supported by the Infectious Diseases and Tropical Medicine Research Center of Shahid Beheshti University of Medical Sciences, Tehran, Iran. Research approval was obtained from the Ethics Committee of Shahid Beheshti University of Medical Sciences (IR.SBMU. RETECH.REC.1398.208).
